# Intraocular Lens Power, Myopia, and the Risk of Nd:YAG Capsulotomy after 15,375 Cataract Surgeries

**DOI:** 10.3390/jcm9103071

**Published:** 2020-09-24

**Authors:** Juha-Matti Lindholm, Ilkka Laine, Raimo Tuuminen

**Affiliations:** 1 Helsinki Retina Research Group, University of Helsinki, 00290 Helsinki, Finland; juha-matti.lindholm@helsinki.fi (J.-M.L.); ilkka.laine@aalto.fi (I.L.); 2 Department of Ophthalmology, Helsinki University Hospital, 00290 Helsinki, Finland; 3 Kymenlaakso Central Hospital, Unit of Ophthalmology, 48210 Kotka, Finland

**Keywords:** cataract surgery, posterior capsule opacification, Nd:YAG capsulotomy, myopia, low-diopter IOL, retrospective cohort study, ZCB00, PCB00, SN60WF, AU00T0, ZA9003

## Abstract

The present study estimated the 5-year cumulative probability of Nd:YAG laser posterior capsulotomy according to the diopter power of implanted hydrophobic acrylic intraocular lenses (IOLs). Data were retrospectively collected of 15,375 eyes having cataract surgery and in-the-bag implantation of hydrophobic acrylic monofocal IOLs at the Ophthalmology Unit of Kymenlaakso Central Hospital, Kotka, Finland between the years 2007 and 2016. The cumulative probability of Nd:YAG capsulotomy was calculated by Kaplan–Meier estimates, and potential risk factors were analyzed using the Cox proportional hazards model. The 5-year cumulative probability of Nd:YAG capsulotomy after cataract surgery was 27.4% (95% confidence interval (CI) 22.9–32.6%) for low-diopter (5–16.5 D) IOLs, 14.6% (13.8–15.5%) for mid-diopter (17–24.5 D) IOLs, and 13.6% (11.7–15.6%) for high-diopter (25–30 D) IOLs. A multivariate Cox regression analysis showed that low-diopter IOLs (HR 1.76; 95% CI 1.38–2.25; *p* < 0.001) were associated with an increased risk of Nd:YAG capsulotomy compared to mid-diopter IOLs over the follow-up period after accounting for other predictors. Real-world evidence shows that low-diopter IOLs are associated with significantly higher risk of Nd:YAG capsulotomy within five years following implantation. Estimation should help in evaluating the risks of cataract surgery in myopic eyes.

## 1. Introduction

Posterior capsular opacification (PCO) due to the proliferation and migration of lens epithelial cells (LECs) remains the most common long-term complication after cataract surgery, although advancements in surgical technology appear to have decreased its incidence [1,2]. The risk factors of PCO have been studied extensively, but the effect of myopia on the incidence of PCO is still not precisely known.

In recent years, several studies have evaluated the effects of intraocular lens (IOL) design and materials on prevention of PCO and subsequent Nd:YAG capsulotomy. Results suggest that a sharp optic edge of the IOL and a firm capsule–IOL adhesion could inhibit LEC migration and PCO [3,4,5]. Myopic eyes are a particular case in this context since they tend to have large capsular bags and are also implanted with thinner IOLs; both are factors that may promote the development of a lower contact pressure between the capsule and IOL. Accordingly, a previous prospective study investigating the capsule–IOL interaction in emmetropic and highly myopic eyes revealed weak capsular adhesion and incompletely adhesive types of capsular bend formation in highly myopic eyes, which appeared to increase the likelihood of PCO during the early 28-day postoperative period [6].

Low-diopter IOLs and myopic eyes provide an opportunity to understand more about the relatively unknown nature of postoperative capsule–IOL interaction, which will play an important role in the development of future IOLs. The aim of our study was to evaluate the real-world 5-year probability of Nd:YAG capsulotomy according to the diopter power of the implanted hydrophobic acrylic monofocal IOL.

## 2. Patients and Methods

This retrospective cohort study was carried out at the Ophthalmology Unit of Kymenlaakso Central Hospital, Kotka, Finland. The study was approved by the Research Director and Chief Medical Officer of the Kymenlaakso Central Hospital and the tenets of the Declaration of Helsinki were followed. We reviewed the registry of operations for phacoemulsification cataract surgeries and Nd:YAG laser capsulotomies between 3 September 2007 and 15 September 2016. All eyes having phacoemulsification surgery and in-the-bag implantation of ZCB00 or preloaded PCB00 (Abbott Medical Optics/Johnson & Johnson Vision Inc., New Brunswick, NJ, USA), SN60WF, or preloaded AU00T0 (Alcon Laboratories Inc, Fort Worth, TX, USA), and ZA9003 IOLs (Abbott Medical Optics/Johnson & Johnson Vision Inc.) were included in the study. Indications for Nd:YAG laser capsulotomy were decreased corrected distance visual acuity and decreased visual function caused by PCO.

Nd:YAG laser capsulotomy was performed with Ellex Super Q YAG laser system (Nova Eye Medical, Adelaide, Australia). Posterior capsule opening was created most often in a cruciate pattern using an Abraham Capsulotomy contact lens, which enhances visualization and focusing. Anesthesia was topical. Patients received postoperative topical IOP-lowering and anti-inflammatory medication according to the physician’s preference.

For group comparisons regarding the baseline demographic and surgical characteristics, continuous and normally distributed data (age) were analyzed with the one-way ANOVA F test, and categorical data (number of patients ≥ 60 years of age, gender, IOL model, and surgeon seniority) with the Pearson’s chi-square test. Post-hoc comparisons among diopter groups were performed with ANOVA pairwise comparisons for the age variable and by identifying statistically significant Pearson’s chi-square adjusted residuals for the categorical variables. Univariate and multivariate Cox proportional hazards regression modeling was used with a stepwise approach to evaluate the effect of dioptric power of implanted IOLs and several other variables including the patient’s age, sex, type of IOL, and the operating surgeon’s seniority on the probability of a Nd:YAG laser capsulotomy event. The IOLs were categorized as low- (5–16.5 D), mid- (17–24.5 D) and high-diopter (25–30 D) lenses [7]. The model included all available eyes, but used a robust estimate of the variance of estimated parameters to account for the correlation between right and left eyes. The cumulative probability of Nd:YAG laser capsulotomy was estimated by Kaplan–Meier survival analysis. The length of the follow-up for each eye was until five years after cataract surgery or until the end of the study period. Statistical analysis was performed using Stata software (version 13.0, StataCorp, College Station, TX, USA). The significance level was set at 5%.

## 3. Results

A total of 17,691 eyes underwent cataract surgery and 1959 Nd:YAG capsulotomies were carried out between 3 September 2007 and 15 September 2016. In all, 15,375 eyes were included in the study according to the inclusion criteria and a total of 1312 Nd:YAG capsulotomies were performed on these eyes during the follow-up. The median duration of follow-up after surgery was 40 months. Baseline demographic and surgical characteristics for all eyes are listed in Table 1.

**Figure 1:**
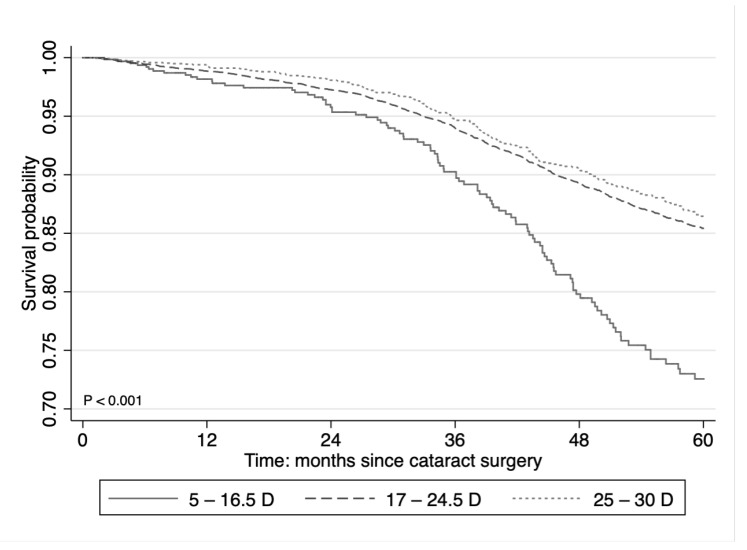
Kaplan–Meier estimates of the Nd:YAG capsulotomy free survival probability according to the diopter power of implanted intraocular lens. D = diopter power.

The cumulative probability of Nd:YAG capsulotomy at five years after cataract surgery was (95% CI 22.9–32.6%) for low-diopter (5–16.5 D) IOLs, 14.6% (95% CI 13.8–15.5%) for mid-diopter (17–24.5 D) IOLs, and 13.6% (95% CI 11.7–15.6%) for high-diopter (25–30 D) IOLs (Figure 1).

Univariate and multivariate Cox proportional hazards regression analyses of potential risk factors associated with Nd:YAG capsulotomy are shown in Table 2. Implantation of low-diopter IOLs was associated with a 76% increase in the hazard of Nd:YAG capsulotomy (HR 1.76; 95% CI 1.38–2.25; *p* < 0.001), compared to mid-diopter IOLs after accounting for other predictors. Patient age younger than 60 years (HR 1.41; 95% CI 1.14–1.73; *p* = 0.001) and female sex (HR 1.21; 95% CI 1.06–1.38; *p* = 0.006) were also associated with an increased risk of Nd:YAG capsulotomy over the follow-up period, whereas implantation of SN60WF/AUT00T0 (HR 0.63; 95% CI 0.56–0.72; *p* < 0.001) or ZA9003 (HR 0.53; 95% CI 0.44–0.65; *p* < 0.001) in comparison with ZCB00/PCB00 IOLs was associated with a decreased risk.

## 4. Discussion

The current study showed that the need for Nd:YAG capsulotomy to treat PCO was approximately two times higher for patients implanted with low-diopter IOLs compared to higher diopter IOLs. Although the overall incidence of PCO has decreased with advancements in modern cataract surgery, some patient groups are at higher risk. Our results support the conclusion of a previous prospective study that the risk of PCO may be higher in myopic eyes because of weak and incomplete capsule adhesion to the IOL [6].

It is widely recognized that myopia is associated with the development of nuclear cataract [8,9]. However, knowledge of the effect of myopia on PCO is inconclusive. In some studies, a high incidence of PCO or Nd:YAG capsulotomy in moderately or highly myopic eyes has been observed [10,11]. A previous small prospective trial concluded that axial myopia did not significantly increase the area or incidence of PCO at four years, although the percentage of eyes with PCO behind the central 3.0 mm zone of the posterior capsule was higher in the myopia group [12]. Varied results in published studies can be at least partially attributed to different definitions of myopia such as based on certain axial length, preoperative refraction, or the diopter power of the IOL implanted. The low-diopter IOL group in our study was defined according to an estimation of the IOL power in eyes with axial length ≥25.2 mm and the available diopter range of the included IOLs [7,13]. In another previous study assessing the refractive effects and incidence of complications of refractive lens exchange, high myopia was defined by implantation of an IOL with a power < 11.0 D [14].

Several mechanisms responsible for PCO formation in myopic eyes have been suggested. Certain growth factors present in the aqueous humor of pathologically myopic eyes have been proposed to influence the development of PCO [12]. There is considerable evidence that firm contact between the capsule and IOL inhibits the migration of LECs [15]. Previous studies on IOL design and material have demonstrated a reduced risk associated with sharp-edged hydrophobic acrylic IOLs [4,16]. This supports the idea that the contact pressure between the optic edge and the posterior capsule is an important factor inhibiting LECs migration. Zhao et al. showed that weak capsular adhesion and incompletely adhesive types of capsular bend at the optic edge during the early postoperative period were more common in highly myopic eyes compared to emmetropic eyes [6]. Highly myopic eyes have large capsular bags and these eyes are also implanted with thinner low-diopter IOLs [17]. Low-diopter IOLs also have reduced posterior convexity. Weak capsular apposition of the IOL could therefore increase the incidence of PCO in myopic eyes.

In a multivariate analysis, younger age, female gender, and implantation of ZCB00/PCB00 IOLs increased the risk of capsulotomy. These results are consistent with the findings of previous studies [2,18,19,20,21]. Differences in health behavior across age groups and by gender may explain the findings. Risk differences between the hydrophobic IOLs can be attributed to the effects of IOL design and biomaterial composition.

There are several strengths and weaknesses related to this study. The study has a large sample size, which is representative of the population and the length of follow-up was sufficiently long. The retrospective cohort design allows for the assessment of the natural history of the disease as well as temporal relationships of risk factors and outcomes. The study also focused on commonly used IOLs. Thus, the results of this study offer real-world clinical practice evidence. The findings are relevant because myopic eyes are already at increased risk for retinal detachment just after cataract surgery alone, and with laser capsulotomy, that rate will probably increase [22]. By also considering the other risks of laser treatment such as IOL damage and cystoid macular edema, and its economic burden to health services and society, finding preventive measures reducing the incidence of PCO and subsequent Nd:YAG capsulotomy would be helpful [23,24].

This study also has potential limitations. In relation to the retrospective design, the results may be affected by some unknown confounding factors and there may be gaps in the study data. Some common systemic and ocular comorbidities were not accounted for in the analysis. The completeness of patient follow-up cannot be fully ascertained because, for example, a patient may have moved out of the region during follow-up. Some patients could also have sought PCO treatment elsewhere instead of the hospital clinic, which would lead to an underestimation of the likelihood of the Nd:YAG capsulotomy. However, we estimate that this proportion is small given the funding of the national healthcare system, physician care practices, and the health services available in the region. Eyes with refractive myopia attributed to the cornea or lens, rather than axial myopia, may not exhibit weak postoperative capsular apposition of the IOL. Some of the patients with high or moderate preoperative myopia may have been operated with a target of mild myopia (up to −2.5 D), which enables the patients to maintain a near-sighted lifestyle. Myopic target refraction requires a slightly higher powered IOL, which could have resulted in classifying some myopic patients near the 16.5 D threshold into the mid-diopter group. Indications for Nd:YAG capsulotomy in this study were decreased visual acuity and visual function, but the clinician’s threshold for intervention may have been higher in low-diopter group because of the higher risks associated with myopic eyes. Nd:YAG capsulotomy rate is not a direct measure for PCO and is also affected by the patient’s tolerance to PCO-induced symptoms and clinical treatment practices, but nevertheless, provides a functional indicator of morbidity related to PCO.

The postoperative capsule–IOL interaction is an interesting research topic that will help to develop better IOLs for the future. For an accommodating IOL that changes its shape, the capsule bag should remain as elastic as possible after surgery, avoiding fibrotization. Capsular contraction and myopia may also lead to IOL subluxation requiring innovative solutions for repair [25]. Our study on low-diopter IOLs elucidates the relationship of PCO and myopia. Future research should also address the risk–benefit ratio of Nd:YAG capsulotomy, especially in highly myopic eyes including zero-power and minus-power IOLs, and preferably in a prospective randomized study setting. With regard to establishing further epidemiologic evidence of a causal relationship between axial myopia and PCO formation, a dose–response relationship between the two should be demonstrated using methods of direct assessment of PCO severity.

In conclusion, the results of this study showed that the estimated risk of Nd:YAG capsulotomy with low-diopter IOLs is significantly higher compared to higher diopter IOLs. The 5-year real-world results of this study should help in evaluating the risks of cataract surgery in myopic eyes.

## Figures and Tables

**Table 1:** Baseline demographic and surgical characteristics.

Variables	All Eyes	5–16.5 D	17–24.5 D	25–30 D	*p* Value
	*n* = 15,375	*n* = 644	*n* = 12,313	*n* = 2418	
Age (years)	75.2 ± 9.0	69.2 ± 9.5 ^a^	75.4 ± 8.9 ^b^	75.8 ± 8.8 ^b^	**<0.001**
≥60 years, *n* (%)	14,399 (93.7)	533 (82.8) *	11,584 (94.1) *	2282 (94.4)	**<0.001**
Gender					**<0.001**
Female	9792 (63.7)	411 (63.8)	7521 (61.1) *	1860 (76.9) *	
Male	5583 (36.3)	233 (36.2)	4792 (38.9) *	558 (23.1) *	
IOL model					**<0.001**
ZCB00/PCB00	6579 (42.8)	340 (52.8) *	5158 (41.9) *	1081 (44.7)	
SN60WF/AU00T0	7098 (46.2)	244 (37.9)	5748 (46.7)	1106 (45.7)	
ZA9003	1698 (11.0)	60 (9.3)	1407 (11.4) *	231 (9.6)	
Surgeon seniority	*n* = 15,280	*n* = 636	*n* = 12,245	*n* = 2399	**0.045**
Specialist	14,170 (92.7)	604 (95.0)	11,329 (92.5)	2235 (93.2)	
Resident	1112 (7.3)	32 (5.0)	916 (7.5)	164 (6.8)	

Data are given as mean ± SD for continuous variables and absolute numbers (with proportions) for categorical variables. ^a,b^ Different superscripts indicate significant differences between the groups in ANOVA post-hoc pairwise comparisons after Bonferroni correction. * Asterisk indicates statistically significant Pearson’s chi-square adjusted residuals after Bonferroni correction. *p* values ≤ 0.05 were considered significant (in bold).

**Table 2:** Crude and adjusted Cox regression analysis of potential risk factors associated with Nd:YAG capsulotomy.

Risk Factor	Crude HR (95% CI)	*p* Value	Adjusted * HR (95% CI)	*p* Value
Age (years)				
<60	1.55 (1.27–1.89)	**<0.001**	1.41 (1.14–1.73)	**0.001**
≥60	1.00		1.00	
Sex				
Female	1.12 (0.99–1.28)	0.077	1.21 (1.06–1.38)	**0.006**
Male	1.00		1.00	
IOL model				
SN60WF + AU00T0	0.63 (0.55–0.72)	**<0.001**	0.63 (0.56–0.72)	**<0.001**
ZA9003	0.53 (0.44–0.65)	**<0.001**	0.53 (0.44–0.65)	**<0.001**
ZCB00 + PCB00	1.00		1.00	
IOL power (diopters)				
5–16.5	1.92 (1.52–2.44)	**<0.001**	1.76 (1.38–2.25)	**<0.001**
17–24.5	1.00		1.00	
25–30	0.89 (0.75–1.06)	0.192	0.85 (0.71–1.01)	0.062
Surgeon				
Specialist	1.22 (0.90–1.66)	0.193		
Resident	1.00			

* Final multivariate Cox regression model with stepwise approach included 15,280 observations. *p* values ≤ 0.05 were considered significant (in bold). HR = hazard ratio.
